# Optimizing laying hen diets: effect of partial maize meal replacement with wheat bran or biscuits crumbs and *Bacillus amyloliquefaciens* CECT 5940

**DOI:** 10.3389/fvets.2025.1557640

**Published:** 2025-08-18

**Authors:** Albertina Felizardo Manteiga, Abilio Paulo Changule, Dercia Hilario Magaia, Nilza Jorge Manjate, Florentina Domingos Chilala, Leonel António Joaquim, Eunice Justino Chivale, Filomena Dos Anjos, Otilia Henrique Tamele Tomo, Manuel Garcia-Herreros, Custodio Bila

**Affiliations:** ^1^Department of Animal and Public Health, Faculty of Veterinary Medicine, Eduardo Mondlane University (UEM), Maputo, Mozambique; ^2^Center for Genetic Resources and Animal Assisted Techniques (CRGTRA), Directorate of Animal Science (DCA), Agricultural Research Institute of Mozambique (IIAM), Matola, Mozambique; ^3^Department of Pharmacy, School of Pharmacy, Federal University of Ouro Preto, Ouro Preto, Brazil; ^4^Department of Research and Development, Intermed Mozambique Lda, Maputo, Mozambique; ^5^Laboratory of Biotechnology and Medicine of Amazonian Animals, Institute of Veterinary Medicine, Federal University of Pará (UFPA), Castanhal, Brazil; ^6^Agricultural Research Institute of Mozambique (IIAM), Angonia, Mozambique; ^7^Veterinary Medicine Institute, Federal University of Pará (UFPA), Castanhal, Brazil; ^8^Department of Animal Production and Food Technology, Faculty of Veterinary Medicine, Eduardo Mondlane University, Maputo, Mozambique; ^9^Section of Animal Nutrition, Faculty of Veterinary Medicine, Eduardo Mondlane University, Maputo, Mozambique; ^10^Directorate of Animal Science (DCA), Agricultural Research Institute of Mozambique (IIAM), Maputo, Mozambique; ^11^National Institute for Agricultural and Veterinary Research (INIAV), Santarém, Portugal; ^12^CIISA-AL4AnimalS, Faculty of Veterinary Medicine, University of Lisbon, Lisbon, Portugal; ^13^Center of Excelence in Agri-Food Systems and Nutrition (CEAFSN) - Eduardo Mondlane University (UEM), Maputo, Mozambique; ^14^Faculty of Veterinary Medicine and Animal Science, Save University (UniSave), Chongoene, Mozambique

**Keywords:** laying hens, *Bacillus amyloliquefaciens* CECT 5940, alternative feed ingredients, nutritional performance and poultry, nutrition

## Abstract

**Background:**

Wheat bran (WB) and biscuit crumbs (BC) offer alternative feed sources for laying hens, potentially improving productivity and economic efficiency.

**Objective:**

This work evaluated the partial replacement of maize meal with WB or BC, with or without the addition of *Bacillus amyloliquefaciens* CECT 5940, on the productive and economic performance of laying hens.

**Methods:**

Five treatments were allocated as follows: T1: a basal diet; T2: a basal diet where 20% of maize meal was replaced by WB; T3: a basal diet that included 20% of WB plus *B. amyloliquefaciens* CECT 5940; T4: a basal diet where 20% of maize meal was replaced by BC; and T5: a basal diet that included 20% of BC plus *B. amyloliquefaciens* CECT 5940. Productive parameters (live weight, laying rate, feed conversion per egg mass, feed conversion per dozen eggs, feed consumption, and viability) and economic metrics (feed costs, production cost per egg, production cost per dozen eggs, gross revenue, gross added value, profitability index, contribution margins, and break-even point) were assessed using ANOVA and the Tukey test.

**Main findings:**

A statistically significant (*p* < 0.05) increase was observed in the LR and FC/dz when maize meal was partially replaced with BC. While the addition of *B. amyloliquefaciens* CECT 5940 to WB significantly improved LR and FC/dz when compared to T2 (*p* < 0.05), no significant difference was seen for BC (*p* > 0.05). As for the economic evaluation, both T2 and T3 significantly reduced production costs (*p* < 0.05). Compared to T1, the partial replacement of maize meal with WB significantly reduced (*p* < 0.05) gross revenue. The addition of *B. amyloliquefaciens* CECT 5940 significantly increased (*p* < 0.05) gross value added, profitability index, and contribution margins, and significantly reduced the break-even point (*p* < 0.05) in comparison with T1 for WB and BC both with (T3 and T5) and without *B. amyloliquefaciens* CECT 5940 (T2 and T4).

**Conclusion:**

It was concluded that the addition of *B. amyloliquefaciens* CECT 5940 to both WB and BC diets was as efficient as the basal diet in terms of productivity and improved economic performance.

## 1 Introduction

The global population continues to grow, leading to an increasing demand for food, especially protein sources. This intensifies competition between humans and livestock, particularly poultry, for staple grains such as maize, which are essential components in both human and animal diets. Poultry, due to its feed efficiency and lower environmental impact compared to other livestock, is increasingly recognized as a key protein source. However, the growing demand for poultry meat further elevates pressure on maize supplies, creating economic and sustainability challenges in feed formulation (1, 2). To address these challenges, there is a growing interest in identifying cost-effective, locally available alternatives to maize that can sustain or enhance poultry performance. The sole aim of replacing maize in poultry diets is to reduce feed costs and alleviate competition with human food chains particularly in resource-constrained settings without compromising animal productivity. Among the alternatives, wheat bran (WB) and biscuit crumbs (BC), both agro-industrial by-products, present promising options for sustainable and economical feed formulation (2, 3).

WB, with an annual global production of around 150 million tons, is a major milling industry by-product rich in protein, starch, lipids, minerals, and minor components such as organic acids and phenolic compounds (2). Although cheaper than maize, WB’s application in monogastric feed is limited by its high fiber and low energy content. However, its nutritional limitations can be addressed through dietary supplementation with probiotics like *Bacillus amyloliquefaciens* CECT 5940, which improve nutrient digestibility and gut health. Similarly, BC derived from surplus or broken biscuits is rich in digestible carbohydrates and energy, and contains moderate amounts of protein (8–10%), fats, and sugars. It is palatable, enhances feed intake, and supports circular economy practices by reducing food waste (4). Prior research has shown that partial replacement of maize with WB or BC can maintain or improve productivity indicators. For instance, Ahmad et al. (3) found that the partial replacement of maize with WB in layer diets led to comparable egg production rates and improved feed conversion ratios. In another study, the inclusion of BC in broiler diets was shown to enhance weight gain and feed efficiency (5). However, the use of such alternative feedstuffs often presents nutritional limitations such as the high fiber content of WB and the relatively low protein content of BC which can impair nutrient absorption and overall performance. To overcome these drawbacks, dietary supplementation with probiotics has gained attention. Previous studies have shown that including probiotics, can enhance nutrient utilization and offset the anti-nutritional effects of unconventional feed ingredients. For instance, Zang et al. (43) and Azzam et al. (6) demonstrated that supplementing high-fiber or low-quality diets with *B. amyloliquefaciens* improved growth performance, gut morphology, and nutrient digestibility in poultry. Similarly, Teng et al. (7) and Abou-Elkhair et al. (44) reported enhanced performance and gut health in layers fed non-conventional diets with added probiotics. These findings justify the inclusion of *B. amyloliquefaciens* in this study, particularly in diets incorporating WB or BC.

*Bacillus amyloliquefaciens* CECT 5940 is a well-documented probiotic strain that produces enzymes such as cellulase, proteases, *α*-amylases and bacteriocins such as barnase and subtilin that enhance nutrient absorption and suppress harmful gut microbes (8, 9). Several studies have demonstrated its benefits in improving digestive health and performance in poultry (6, 10, 43). However, most of these findings stem from laboratory settings and often do not assess economic implications or field-level performance. Given the rising costs of conventional feed ingredients, the economic benefits of incorporating WB and BC particularly in combination with probiotics merit further investigation. Both ingredients are generally more affordable and accessible than maize, especially in regions with milling and biscuit production industries, potentially offering smallholder farmers a practical means of reducing production costs and improving profitability (11).

While prior studies have assessed the nutritional effects of WB and BC, there remains a gap in understanding their combined use with probiotics like *B. amyloliquefaciens* in real-world laying hen operations. This study aims to fill that gap by evaluating whether partially replacing maize meal with WB or BC, with or without the addition of *B. amyloliquefaciens* CECT 5940, can sustain or improve laying performance and reduce feed costs under field conditions. We hypothesized that these dietary strategies would enhance egg production, feed conversion efficiency, and economic performance, thereby offering a sustainable and cost-effective alternative to conventional maize-based diets.

## 2 Materials and methods

### 2.1 Ethics statement

This study was conducted following the ethics of animal experimentation recommendations of the National Research Council of Mozambique. The research protocol was reviewed and approved by the Institutional Animal Care and Use Committee of Eduardo Mondlane University, Mozambique (Record no. IACUC-EMU-10/2023). All procedures followed ethical standards, prioritizing animal welfare during the study. This included provisions for proper housing, feeding, and monitoring for health issues.

### 2.2 Animals and experiment location

The experiment lasted for 45 days and involved 400 Lohman Brown hens that had been actively laying eggs for a period of 30 weeks, specifically from July to September 2023, prior to the start of the study. During the study period, ambient temperatures in Marracuene ranged from approximately 15°C to 25°C, which falls within the thermoneutral zone for laying hens and is considered suitable for optimal performance. The hens were kept in a battery cage system, which included a deep pit for housing with access to natural ventilation. Illuminated by a combination of artificial and natural light sources, the study was conducted at the animal farm operated by InterMed Mozambique Lda, located in Marracuene. The district of Marracuene is located 30 km north of Maputo and is part of the Maputo Province in Mozambique. It is situated at latitude of approximately 25.8976° S and 32.6744° E, with an elevation of about 26 meters (85 feet) above sea level. The predominant climate in Marracuene is tropical savanna, influenced by its proximity to the sea conducive for poultry farming. Temperatures are warm, averaging above 20°C, with an annual temperature variation of less than 10°C. Relative humidity varies from 55 to 75%, and rainfall is moderate, with an average annual total ranging from 500 mm inland to 1,000 mm along the coast. The rainy season occurs from October to April, with 60 to 80% of the precipitation concentrated in the months of December to February (45).

### 2.3 Experimental design

In a fully randomized study design, 400 laying hens were categorized into five groups, with 4 replicates per treatment, and 20 birds per replicate, totaling 80 birds per treatment group. This randomization was performed to avoid bias, ensuring that each hen had an equal chance of being assigned to any treatment group ensuring equal representation in terms of initial weight and egg production. The hens were randomly allocated to one of the following five treatments. T1: a basal diet; T2: a basal diet where 20% of maize meal was replaced by WB at a rate of 20%; T3: a basal diet that included 20% of WB plus *B. amyloliquefaciens* CECT 5940; T4: a basal diet where 20% of maize meal was replaced by BC; and T5: a basal diet that included 20% of BC plus *B. amyloliquefaciens* CECT 5940 (Evonik, Essen, Germany) at a concentration of 2 × 10^9^ cfu/g and substituted with maize meal. The nutrient content of diets was calculated using standard feed formulation software based on tabulated nutrient values for each ingredient (Table 1). No laboratory proximate analysis was performed. The basal diet, WB, and BC were sourced from the local market.

**Table 1:** Proportions of feed ingredients in the experimental diets.

Ingredients	Calculated composition				
	T1	T2	T3	T4	T5
	(Kg)				
Maize meal	75.00	60.00	60.00	60.00	60.00
Soya bean meal	12.20	12.20	12.20	12.20	12.20
Wheat bran	5.00	20.00	20.00	5.00	5.00
Biscuits crumbs	0.00	0.00	0.00	15.00	15.00
Cottonseed cake meal	4.45	4.45	3.65	4.45	3.65
*B. amyloliquefaciens* CECT 5940	0.00	0.00	0.80	0.00	0.80
Trace mineral premix^1^	3.00	3.00	3.00	3.00	3.00
Vitamin premix^2^	0.10	0.10	0.10	0.10	0.10
Dicalcium phosphate	0.25	0.25	0.25	0.25	0.25
Total	100	100	100	100	100
Calculated composition					
Energy (kcal/kg)	3,265.02	3,284.13	3,265.02	3,213.74	3,214.71
Protein	14.60	14.50	14.50	12.57	13.40
Fiber	3.19	2.95	2.89	2.49	2.57
Ether extract	3.32	3.26	3.23	3.08	2.68
Lysine	2.59	2.62	2.61	2.28	1.82
Methionine	1.86	1.81	1.81	1.64	1.26
Calcium	4.00	4.04	4.04	0.13	0.06
Phosphorus	0.40	0.70	0.70	0.41	0.25

1 Trace mineral premix provided the following per kilogram of diet: Mn, 80 mg; Fe, 60 mg; Zn, 60 mg; Cu, 5 mg; Co, 0.2 mg; I, 1 mg; Se, 0.15 mg; Ca, 446.9 mg.

2 Vitamin premix provided the following per kilogram of diet: vitamin A 12,000 IU; cholecalciferol, 2000 IU; vitamin E, 35 IU; vitamin k_3_, 5 mg; thiamin, 3 mg; riboflavin, 6 mg; niacin, 20 mg; Ca-d-pantothenate, 6 mg; pyridoxine, 5 mg; vitamin B_12_, 15ug, folacin, 0.75 mg, D-biotin, 45 ug, choline chloride, 125 mg; vitamin C, 50 g.

Hens were housed in a battery cage system, with each replicate consisting of five adjacent cages, each containing 4 birds. This allowed for efficient individual feed intake monitoring and egg collection, while maintaining uniform management across all replicates. They had unrestricted access to water, while each hen was provided with 120 g. of feed per day. The leftover feed was weighed to determine daily feed consumption. All groups were treated equally during the entire experiment.

At the beginning of the study, initial body weights and egg production ratios were documented for all experimental hens, and this data was updated weekly. Daily records were kept for egg production, instances of soft eggshells, egg weights, and any mortality events. Additionally, weekly feed consumption was tracked. The weekly calculations included feed intake, egg production ratios, soft eggshell production ratios, feed conversion efficiencies per egg mass and per dozen eggs, as well as mortality rates (12).

### 2.4 Economic analysis

The economic performance of each dietary treatment was assessed using key profitability indicators, based on feed ingredient costs, egg production, and prevailing market prices. The analysis followed a modified approach adapted from Egbetokun et al. (13) and Andriani et al. (14). Feed cost per kilogram ($/kg) was calculated by dividing the total cost of ingredients by the total weight of each diet, while feed cost per hen was determined by multiplying the feed consumed per replicate by the feed cost per kilogram. Production cost per egg was computed as the total feed cost per replicate divided by the number of eggs produced, and cost per dozen eggs was based on the feed required to produce 12 eggs. Gross revenue was calculated by multiplying the total number of eggs produced by the market price per egg, and gross value added was obtained by subtracting total feed costs from gross revenue. The profitability index (%) was the ratio of gross value added to gross revenue, indicating the share of revenue retained after feed costs. Contribution margin ($ and %) reflected the income available to cover fixed costs and profit, while the break-even point represented the revenue required to fully cover feed costs. All monetary values were expressed in US dollars (USD), using local ingredient and egg prices from July to September 2023. These calculations enabled a comparative assessment of the cost-effectiveness and economic viability of each diet under practical production conditions.

### 2.5 Statistical analysis

The data obtained were analyzed using a one-way analysis of variance through SPSS software version 25 (IBM Corp., NY, USA). The Shapiro–Wilk test was used to check for normality, while Levene’s test assessed the equality of variances among groups. After conducting ANOVA, Tukey’s Honestly Significant Difference (HSD) test was applied to make pairwise comparisons between treatment means at a significance level of 5%.

## 3 Results

### 3.1 Impact of incorporating *Bacillus amyloliquefaciens* CECT 5940 into wheat bran and biscuits crumbs based-diets on the productivity of laying hens

The effects of partially replacing maize meal with wheat bran (WB) or biscuit crumbs (BC), with or without *B. amyloliquefaciens* CECT 5940, are presented in Table 2. No significant differences (*p* > 0.05) were observed among treatments for initial or final live weight, weight gain, feed consumption, or viability.

**Table 2:** Effect of partial replacement of corn with or without addition of *B. amyloliquefaciens* CECT 5940 on laying hen productive performance.

Parameters	T1	T2	T3	T4	T5	*p*-value
Initial Live Weight (g)	1,310 ± 0.02 ^a^	1,407 ± 0.02 ^a^	1,582 ± 0.03 ^a^	1,501 ± 0.02 ^a^	1,692 ± 0.02 ^a^	0.083
Final Live Weight (g)	1,473 ± 0.02 ^a^	1,563 ± 0.01 ^a^	1,742 ± 0.02 ^a^	1,654 ± 0.01 ^a^	1,845 ± **0.01** ^a^	0.076
Weight Gain (g)	163 ± 1.11 ^a^	156 ± 2.44 ^a^	160 ± 1.83 ^a^	153 ± 1.81 ^a^	153 ± 1.81 ^a^	0.872
FC (kg)	5.10 ± 0.05^a^	5.13 ± 0.06^a^	5.13 ± 0.06 ^a^	5.12 ± 0.05 ^a^	5.12 ± 0.05 ^a^	0.899
LR (%)	66.66 ± 12.93^a^	49.78 ± 12.65^b^	70.66 ± 7.79^a^	73.78 ± 3.56^a^	73.11 ± 10.04^a^	0.041
EM	0.27 ± 0.09^a^	0.21 ± 0.04^a^	0.32 ± 0.05^ab^	0.37 ± 0.08^b^	0.35 ± 0.06^b^	0.046
FC/EM (kg)	2.10 ± 1.12^a^	2.36 ± 0.44^a^	1.51 ± 0.24^b^	1.34 ± 0.29^b^	1.40 ± 0.26^b^	0.021
FC/dz (kg)	1.12 ± 0.25^a^	1.52 ± 0.37^b^	1.02 ± 0.10^a^	0.98 ± 0.04 ^a^	1.00 ± 0.16 ^a^	0.033
Vb (%)	100	100	100	100	100	-

Mean ± S. E. M. Different letters within the same row (a, b) represent statistical differences among treatments within the same parameter (*p* ≤ 0.05). LW, Live weight of the layers; FC, Feed consumption; LR, Laying rate; EM, Egg mass; FC/EM, Feed conversion per egg mass; FC/dz, Feed conversion per dozen; Vb, Viability of the layers. T1: Commercial feed; T2: inclusion of 20% WB, T3 inclusion of 20% WB with probiotic *B. amyloliquefaciens* CECT 5940; T4: inclusion of 20% BC, T5 inclusion of 20% BC with probiotic *B. amyloliquefaciens* CECT 5940.

Laying rate (LR) was significantly reduced (*p* < 0.05) in the 20% WB group (T2: 49.78 ± 12.65%) compared to the control (T1: 66.66 ± 12.93%). However, supplementing WB with *B. amyloliquefaciens* (T3) improved LR (70.66 ± 7.79%) to levels statistically similar to T1.

BC diets (T4 and T5) significantly increased egg mass compared to T2 (*p* < 0.05), but were statistically similar to T1. Feed conversion per egg mass (FC/EM) and per dozen eggs (FC/dz) were also significantly improved in T3, T4, and T5 compared to T2 (*p* < 0.05) (Table 2).

### 3.2 Cost–benefit analysis resulting from the incorporation of *Bacillus amyloliquefaciens* into WB and BC-based diets for laying hens

Replacing maize meal with 20% WB (T2) and BC (T4) significantly decreased feed production costs per kilogram to $0.33, compared to $0.55 in the basal diet (T1) (*p* < 0.05). Treatments with *B. amyloliquefaciens* addition (T3 and T5) also maintained this reduced feed cost, indicating that probiotic inclusion did not increase feed expenses (Table 3).

**Table 3:** Effect of partial substitution of maize with WB or BC, without or with *B. amyloliquefaciens* CECT 5940 on production cost parameters.

Parameters	T1	T2	T3	T4	T5	*p* values
Cost of producing feed ($/kg)	$0.55\pm 0.02^{a}$	$0.33\pm 0.03^{b}$	$0.33\pm 0.03^{b}$	0.33 ±0.03 ^b^	0.33 ±0.03 ^b^	0.001
Cost of feed ($)	$5.99\pm 0.05^{a}$	$3.55\pm 0.13^{b}$	$3.60\pm 0.04^{b}$	3.60 ±0.04 ^b^	3.74 ±0.13 ^b^	0.003
Cost of egg production ($)	$0.05\pm 0.01^{a}$	$0.04\pm 0.01^{ab}$	$0.04\pm 0.01^{ab}$	0.03 ±0 .00^b^	0.03 ± 0.00^b^	0.005
Cost of egg production/ Dz ($)	$0.61\pm 0.14^{a}$	$0.50\pm 0.12^{a}$	$0.42\pm 0.13^{ab}$	0.33 ±0 .02^b^	0.35 ±0.06 ^b^	0.004

Means ± S. E. M. Different letters within the same row (a-c) represent statistical differences among treatments within the same parameter (*p* ≤ 0.05), T1: Commercial feed; T2: inclusion of 20% WB, T3 inclusion of 20% WB with probiotic *B. amyloliquefaciens* CECT 5940, T4: inclusion of 20% BC, T5 inclusion of 20% BC with probiotic *B. amyloliquefaciens* CECT 5940, $, United States Dollar.

The reduction in overall feed costs translated to decreased production costs per egg: $0.04 for T2 and T3 (WB diets) and $0.03 for T4 and T5 (BC diets), compared to $0.05 in T1 (*p* < 0.05). Similarly, the cost of production per dozen eggs was reduced across treatments, with T4 achieving the lowest at $0.33, versus $0.61 in the basal diet (*p* < 0.05).

While feed costs decreased, no significant differences were observed in total egg mass or feed conversion per egg mass between BC diets and T1 (*p* > 0.05). However, laying rate and FC/dz improved significantly with BC diets (*p* < 0.05). The addition of *B. amyloliquefaciens* did not significantly alter production costs compared to non-probiotic diets.

### 3.3 Profitability analysis resulting from the incorporation of *Bacillus amyloliquefaciens* into WB and BC-based diets for laying hens

Economic profitability metrics derived from Table 4 reveal that replacing maize with WB (T2) lowered gross revenue ($6.95) compared to T1, whereas probiotic addition (T3) increased gross revenue to $9.92. BC diets (T4 and T5) generated higher gross revenues ($10.39 and $10.12, respectively).

**Table 4:** Effect of partial substitution of maize with WB or BC, without or with *B. amyloliquefaciens* (T3) on profitability parameters.

Trat.	Gross revenue ($)^1^	Gross value added ($)^2^	Profitability index (%)^3^	Contribution margin ($)^4^	Contribution margin (%)^5^	Break-even point ($)^6^
T1	9.39 ± 1.82^a^	3.47 ± 1.82^a^	34.73 ± 14.80^a^	0.67 ± 0.07^a^	67.36 ± 7.40^a^	8.88 ± 1.10^a^
T2	6.95 ± 1.85^b^	3.40 ± 1.85^a^	45.9 ± 14.23^ab^	0.73 ± 0.06^a^	73.34 ± 6.64^a^	4.87 ± 0.45^b^
T3	9.92 ± 1.12^a^	6.24 ± 1.12^b^	62.52 ± 3.69^b^	0.81 ± 0.02^b^	81.32 ± 1.81^b^	4.53 ± 0.10^b^
T4	10.39 ± 0.50^a^	6.79 ± 0.50^b^	65.25 ± 1.71^b^	0.82 ± 0.08^b^	82.62 ± 0.85^b^	4.36 ± 2.90^b^
T5	9.92 ± 1.62^a^	6.18 ± 1.62^b^	61.32 ± 7.63^b^	0.82 ± 0.03^b^	81.51 ± 3.05^b^	4.59 ± 0.18^b^
*p* values	0.000	0.000	0.000	0.000	0.000	0.000

Means ± S. E. M. Different letters within the same column (a, b) represent statistical differences among treatments within the same parameter (*p* < 0.05). T1: Commercial feed; T2: inclusion of 20% WB, T3: inclusion of 20% WB with probiotic *B. amyloliquefaciens* CECT 5940, T4: inclusion of 20% BC, T5 inclusion of 20% BC with probiotic *B. amyloliquefaciens* CECT 5940, $, United States Dollar.

1 Gross revenue = Total eggs × price per egg.

2 Gross value added = Gross revenue − feed cost.

3 Profitability index = (Gross value added ÷ Gross revenue) × 100.

4 Contribution margin = Revenue − variable cost.

5 Contribution margin = Revenue − variable cost.

6 Break-even point = Feed cost ÷ price per egg.

Gross value added (GVA) remained similar between T1 and T2 but increased significantly in probiotic and BC diets (*p* < 0.05). The profitability index was significantly higher in all treatments with partial maize replacement, with T4 and T5 achieving 65.25 and 61.32%, respectively, compared to 34.73% in T1 (*p* < 0.05).

The break-even point was lowest in T4 ($4.36), indicating greater economic resilience, followed by T2 ($4.87). These findings suggest that BC diets, especially with probiotic supplementation, enhance profitability and economic sustainability of production.

## 4 Discussion

The use of alternative feed sources for laying hens offers a promising approach to alleviating the high dependence on imported ingredients and environmental pressures linked to the current climate change issues, especially for low-income countries in the tropics such as Mozambique (11). Therefore, one of the aim of our study was to evaluate the effects of partial replacement of maize meal by WB or BC with the addition of probiotic *B. amyloliquefaciens* CECT 5940 on the average laying hen live weight (LW), egg-laying rate (LR), egg mass (EM), feed conversion per dozen eggs (FC/dz), and laying hen viability (Vb). Notably, combinations of alternative ingredients with the addition of probiotics have been studied on poultry nutrition and are well known individually but their effects combined especially on laying hens are still a novelty (9).

The present study explored the impact of partially replacing maize meal in the diets of laying hens with alternative ingredients, specifically wheat bran (WB) and biscuit crumbs (BC), and examined the efficacy of including the probiotic *Bacillus amyloliquefaciens* CECT 5940. It aimed to evaluate not only the productive outcomes but also the economic feasibility of these dietary changes.

Our study revealed that replacing maize meal using a combination of 20% of WB and *B. amyloliquefaciens* CECT 5940 added at 2 × 10^9^ cfu/g to the diet did not adversely affect laying hens’ performance. Due to the similar responses in egg production, feed efficiency and egg quality, these findings suggest that replacement of laying hen diets up to 20% is within the estimated range if diets are adjusted for minerals and vitamins.

In our study, the inclusion of WB at 20% did not significantly modify live weight, feed consumption, or viability, but it correlated with a decline in laying rate (LR) and feed conversion per dozen eggs. These results align with findings from Kamal et al. (15), who suggested that high fiber content in WB might limit energy density, impacting egg production. Interestingly, the addition of *B. amyloliquefaciens* added at 2 × 10^9^ cfu/g in the WB diet (T3) ameliorated these metrics, restoring them to levels comparable with the control diet (T1). This reinforces the idea that probiotics can enhance gut health and nutrient absorption, potentially offsetting the negative impacts associated with high fiber diets (46). Moreover, Balasubramanian et al. (47), suggested that probiotics in the diets can produce enzymes such as proteases, lipases, and amylases. In addition, Bai et al. (16), Gadde et al. (17), Oh et al. (18) and Qiu et al. (48), referred that *Bacillus* spp. increases the antioxidant capacity under various stress conditions and increases immunity. This could be the reasons why no negative effects were seen with the replacement of maize in the diet especially regarding layers viability, which is very important parameter when testing feed alternatives.

Conversely, the replacement of maize meal with BC (T4) demonstrated notable improvements. The 20% BC treatment (T4) enhanced LR and feed conversion significantly in comparison to WB diet (T2), verifying previous research by Kholif et al. (19), which posits that BC’s digestibility and nutrient profile provide a superior energy source in poultry diets compared to traditional grains. Moreover, the combination of BC with *B. amyloliquefaciens* (T5) further supported productive performance, indicating a synergistic effect that warrants further investigation.

According to our findings, the laying hens surprisingly experienced an increase in live weight (LW). Zhang et al. (20) stated that WB is a moderate source of insoluble dietary fiber, which has shown to be beneficial to nutrient utilization by improving the physiology of the gastrointestinal tract. These authors also indicated that supplementation with WB enhanced nutrient digestibility by improving antioxidant status, gizzard development, intestinal digestive enzyme activities and morphology in broilers. This could explain the increase in the LW in this study. On the other hand, a decline on egg-laying rate, egg mass and feed conversion per dozen eggs was observed when the basal diet was partly replaced with WB diet without the probiotic *B. amyloliquefaciens* CECT 5940; however, when the probiotic was added, the performance ameliorated in all these parameters. There was a significant effect on laying rate and feed conversion when *B. amyloliquefaciens* CECT 5940 was added compared to WB without the probiotic. This enhancement in laying performance could be attributed to the probiotic’s role in promoting gut health, which may lead to better nutrient absorption and overall hen productivity.

These findings align with previous studies indicating that probiotics can enhance the performance of poultry by improving gut morphology and nutrient digestibility (21). While the feed conversion efficiency showed variability among treatments, the positive trend observed when *B. amyloliquefaciens* was included suggests that probiotics can serve as a valuable dietary additive to optimize the performance of laying hens fed high-fiber diets, such as those containing WB. Studies by Forte et al. (22), Guo et al. (23) and Wang et al. (24) have shown that adding different types and doses of *Bacillus* to the diets can increase feed intake and laying performance. Zhang et al. (25) and (26) also found that average daily gain was improved by the supplementation of *Bacillus*-based probiotics dietary as compared to those of the controls (27). Similar results were described by Amerah (28) who found that dietary supplementation with *B. subtilis* at 1.5 × 10^8^ cfu/kg in diets could improve the feed conversion ratio by reducing the feed intake. However, when compared the feed conversion between basal diet and partial replacement of maize meal with WB supplemented with *B. amyloliquefaciens* CECT 5940 no significant effect was seen. Mountzouris et al. (29), Lee et al. (30) and Cufadar et al. (31) reported that dietary probiotics had minimal or no effects on the growth performance (29, 30). Lee et al. (30) and Zhang et al. (25) refer that the results can differ depending on the strains of probiotics, administration dosage, methods of preparation, bird age, diet composition, and hygiene status. Still regarding laying rates, the probiotic addition to WB diet (T3) showed a significant effect when compared to T2, as the laying rate experienced a rise. However, no statistically significant difference was seen in comparison with the basal diet (T1), meaning that replacing maize meal with WB up to 20% with *B. amyloliquefaciens* CECT 5940 added has no harmful effects on eggs laying. Agreeing with our results, Castañeda et al. (32) and Oketch et al. (33), also found a significant effect on laying rates when hens were fed with a diet containing *Bacillus* strains probiotics.

Similarly, when substituting maize meal with BC, the partial replacement of maize meal did not significantly impact total egg mass or feed conversion per egg mass. These findings agree with those observed by Olafadehan et al. (49). However, it led to a statistically significant increase in the laying rate and improved feed conversion per dozen eggs. This indicates that BC can be a viable alternative to maize meal in layer hen diets without negatively affecting overall egg production. Feed cost is the most limiting factor in laying egg production systems (34). In order to maximize profits, it is necessary to keep production costs as low as possible. Considering the cost-effectiveness of the WB and *B. amyloliquefaciens* CECT 5940 supplementation diets at 2 × 10^9^ cfu/g, the economic evaluation data showed promising results for modern laying egg production. The partial replacement of maize meal with WB or BC significantly reduced production costs across various metrics, including feed production costs, egg production costs, and costs per dozen eggs when compared to the basal diet (T1). Notably, WB and BC diets with or without the probiotic reported a substantial decrease in feed costs, reflecting the economic viability of incorporating lower-cost feed ingredients. The profitability index increased for all treatments, indicating a favorable return on investment compared to the control diet (T1). The price of the feed obtained from the accumulated consumption was estimated to be very economical as it represented a reduction in the production cost of the feed per kilo when WB and BC were supplemented with *B. amyloliquefaciens* CECT 5940.

Considering that WB, although being recognized by its high dietary fiber and phytochemical contents, providing excellent physiological effects for birds, it is a by-product of dry milling of wheat (35). Therefore, it is much cheaper and widely available than whole wheat and maize meal, the main raw materials commonly used to feed layers. Thus, substituting any of these ingredients with WB leads to an instantly cost decrease (36). Similar studies showed significant economic benefits when basal diets were partly replaced by alternative ingredients. An interesting finding is regarding the addition of *B. amyloliquefaciens* CECT 5940 to WB treatment, which had a significant reduction on cost of egg production/Dz compared to both basal diet (T1) and partial replacement of maize meal without the probiotic (T2). This could be explained as a result of the increase in laying rate (LR) while at the same time the feed conversion/dz (FC/Dz) reduced when the layers were fed with the *B. amyloliquefaciens* CECT 5940 based diet in T3 (Table 2). This means that when LR increases and FC/Dz decreases the production system is sustainable and therefore is better cost-effective. Similar results were described by Poberezhets and Kupchuk (37) who found that the use of probiotics in the diet of broiler chickens reduced feed costs. Gomes et al. (50) also observed reductions in feed costs for piglets aged 43 to 67 days with the inclusion of rice by-products in the diet. While the cost of egg production per dozen eggs reduced by up to 0.11$ with WB without the probiotic in comparison to the basal diet, adding *B. amyloliquefaciens* CECT 5940 reduced the cost by 0.07$ compared to the treatment (T2). Rufino et al. (51) stated that with the positive effect obtained from the inclusion of alternative feeds, small producers who do not have the capital to purchase maize meal and concentrates will benefit and will be able to maintain good production.

The economic analysis showcased that incorporation of WB and BC led to notable reductions in feed and egg production costs per kilogram. Previous studies have similarly illustrated the cost-saving potential of fiber-rich feedstuffs in poultry diets (38). However, while WB reduced production costs, the overall performance metrics suggest that BC remains a more economically viable option. The evaluation demonstrated that groups with BC yielded the highest gross revenue ($10.39), outpacing WB treatments. These metrics reflect a highly favorable return on investment and suggest that biscuit crumbs are the most economically viable replacement tested. The profitability index improved substantially in T3, suggesting that the inclusion of probiotics not only enhances production efficiency but also maximizes profitability per dollar invested. This demonstrates the economic relevance of using probiotics to mitigate performance losses in fiber-rich diets. The observed improvements in contribution margins further emphasize the financial benefits of using probiotics in conjunction with alternative feed sources.

Regarding the break-even point analysis which is the total revenue equal to the total cost or the same profit, where the business has not yet made a profit or is equal to zero (39); and contribution margin analysis, which shows the valuation of the product, highlighting the extent to which it is capable of contributing to profits (40), our study shows that overall, the partial substitution of maize meal with or without the addition of *B. amyloliquefaciens* CECT 5940 has a beneficial effect on the profitability leading to a significant reduction of break-even point. Our results suggest that these dietary strategies could enhance financial sustainability in poultry operations. This is particularly important for small-scale farmers who may face financial constraints and rely on cost-effective alternatives to standard feed ingredients.

The partial replacement of maize meal with WB (T2) showed that there is a significant reduction in gross revenue. This makes sense because when the basal diet (T1) was replaced by WB (T2) the laying rate experienced a decrease (Table 2), and therefore the amount of eggs available which are the final products decreased as well, resulting in reductions in gross revenue. A different trend was seen when the probiotic was added to WB (T3) resulting in an increase in laying rate leading to a significant increase in gross revenue compared to T2. The parameters such as gross added value, profitability index, and contribution margins improved significantly when WB was added *B. amyloliquefaciens* CECT 5940 compared to the control treatment (T1) whereas the break-even point decreased significantly. These findings mean that production is economically viable and thus the net profit is higher with the addition of *B. amyloliquefaciens* CECT 5940. This may be due to the significant increase that the probiotic caused in the egg-laying rate and improved feed conversion per dozen eggs, which means that less feed was consumed to produce a dozen eggs. In addition, the 20% substitution of maize for WB contributes to reducing production costs and therefore positively affects the contribution margin and the break-even point.

Furthermore, the partial replacement of maize meal with BC, both without and with *B. amyloliquefaciens* CECT 5940, led to increases in contribution margin, gross added value, and profitability index, as well as a reduction in the break-even point. These findings indicate that the use of BC can enhance the overall economic viability and profitability of layer hen production.

By aligning the productive and economic outcomes, it becomes clear that BC due to its high digestibility and low cost is a superior alternative to maize meal. WB, while more challenging due to its fiber content, can still be used effectively when supplemented with *B. amyloliquefaciens* CECT 5940. This synergy enhances both biological and financial performance, as observed in treatment T3. Overall, the findings support the hypothesis that integrating locally available agro-industrial by-products and probiotics can reduce reliance on conventional feed ingredients without compromising hen performance or economic return. This approach holds particular relevance for poultry producers in resource-constrained regions, offering both sustainability and profitability.

## 5 Conclusion

In conclusion, the results of this study demonstrate that the partial replacement of maize meal with WB or BC, along with the addition of *B. amyloliquefaciens* CECT 5940, is as efficient as maize meal-based diets in promoting the performance of laying hens. Moreover, it has proven to be a beneficial, highly cost-effective, and economically viable strategy for improving the productive performance and economic parameters of laying hens, as it significantly contributed to the reduction of all production cost parameters and better economic return achievements.

## Data Availability

The original contributions presented in the study are included in the article/supplementary material, further inquiries can be directed to the corresponding author.
